# Osteoma of the Medial Wall of the Maxillary Sinus: A Primary Cause of Nasolacrimal Duct Obstruction and Review of the Literature

**DOI:** 10.1155/2014/348459

**Published:** 2014-11-11

**Authors:** Athanasios Saratziotis, Enzo Emanuelli

**Affiliations:** ^1^Department of Otolaryngology, Head and Neck Surgery, University Hospital of Larissa, 41110 Larissa, Greece; ^2^Department of Otolaryngology and Otologic Surgery, University Hospital of Padova, 35100 Padova, Italy

## Abstract

A 74-year-old male patient presented to the outpatient department with left-sided epiphora and chronic dacryocystitis, without any history of head trauma or previous nasal or paranasal sinuses surgery. No abnormalities were noted at the time with the use of nasal endoscopy. The computed tomography scan however revealed an osteoma of the medial wall of the left maxillary sinus. An endonasal endoscopic dacryocystorhinostomy (DCR) with osteoma removal by using a drill with temporary silicone stenting of the nasolacrimal duct system was performed. Due to a granuloma formation at the DCR-window site 2 months postoperatively a revision-DCR was performed and the new window remained patent at control 6 months after surgery.

## 1. Introduction

Acquired nasolacrimal duct obstruction (NLDO) may be due to various causes including idiopathic stenosis of the nasolacrimal duct, with or without formation of nasolacrimal duct mucoceles, trauma, or surgery to the paranasal sinuses and tumors of the nasal cavity.

Osteomas are benign tumors developing in the paranasal sinuses and the nasal cavity. They usually grow in the frontal sinus near the nasofrontal duct and, in decreasing order of frequency, in the ethmoid, sphenoid, and maxillary sinus. They usually remain asymptomatic and they tend to be an incidental finding on radiographic studies. Rarely, they extend out of the sinus limits.

To our knowledge, acquired NLDO associated with an osteoma of the medial wall of the maxillary sinus has not been reported in medical literature so far.

Various methods such as laser beams, forceps, curettes, neurosurgical microrongeurs, and drills have been used for the formation of the lateral nasal wall osteotomy during endonasal dacryocystorhinostomy (DCR) [1]. By means of an endoscopic (rather than an external) DCR approach, not only NLDO but also the concomitant endonasal pathology can be simultaneously addressed.

## 2. Case Presentation

**Figure 1:**
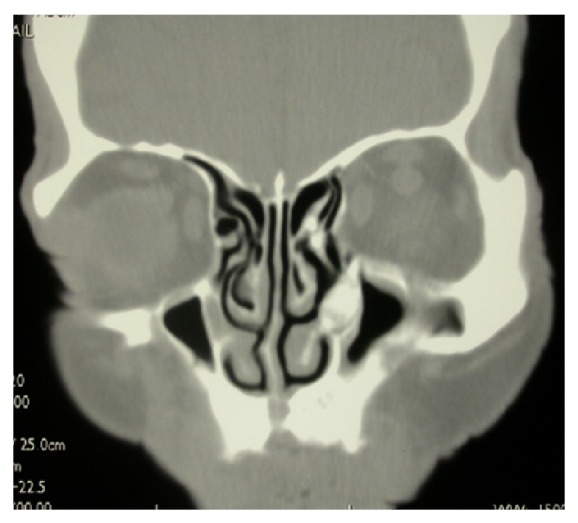
Coronal view of the CT scan of the paranasal sinus showing the osteoma of the medial wall of the left-sided maxillary sinus.

**Figure 2:**
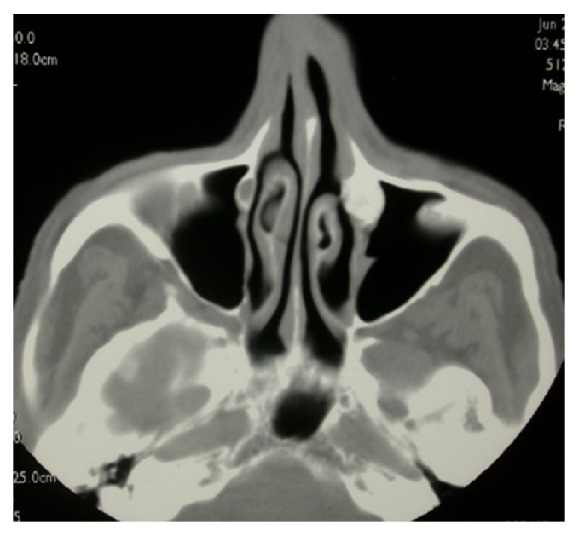
Axial view of the CT scan of the paranasal sinus showing the osteoma of the medial wall of the left-sided maxillary sinus.

**Figure 3:**
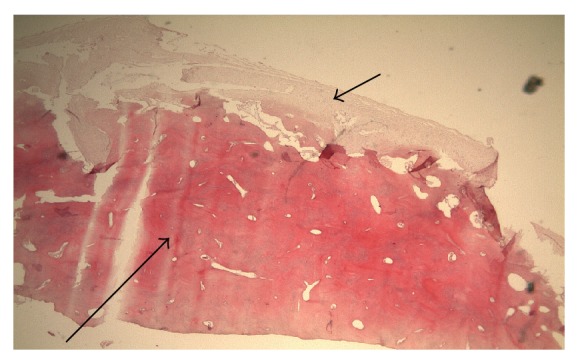
Histological examination (analysis X2,5) of this slow-growing, bone-forming tumor revealed the presence of dense, compact mature bone in paucicellular fibrous stroma. Large trabeculae of mature, lamellar bone can be seen: mature bone (big arrow) and fibrous stroma (small arrow).

**Figure 4:**
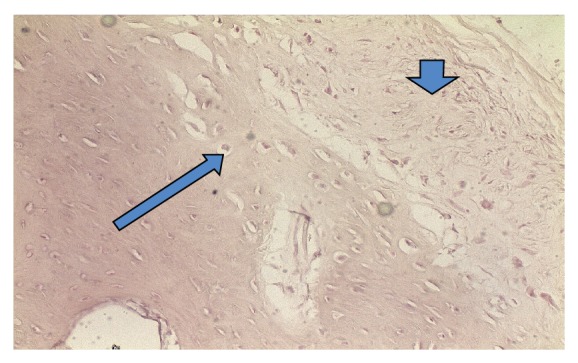
Histological examination of the osteoma (analysis X20): osteocyte (big arrow) and fibrous tissue (small arrow).

A 74-year-old male patient presented to the outpatient department with left-sided epiphora and chronic dacryocystitis, without any record of head trauma or previous surgery of the nose or paranasal sinuses. On nasal endoscopy no abnormalities were noted. A diagnosis of saccal NLDO was made by clinical examination including lacrimal irrigation and fluorescein tests (Jones I and II tests) as well as probing of the canaliculi with a blunt probe up to bony contact with the lacrimal sac fossa performed by an ophthalmologist. The imaging studies (CT scan) revealed a 1 cm osteoma of the ventral half of the medial wall of the left maxillary sinus causing total obstruction at the level of the lacrimal sac (Figures 1, 2, 3, and 4).

Under general anesthesia, an endonasal endoscopic dacryocystorhinostomy (DCR) without flaps [2] using a cutting- and diamond-burr drill as well as the osteoma removal using a cutting-burr drill was performed. A temporary silicone stenting of the nasolacrimal duct system was applied. No postoperative complications occurred. An oral antibiotic therapy (amoxicillin and clavulanic acid 1 gr twice a day) was initiated on the day of surgery and continued for 5 days. A concomitant topical therapy with antibiotic and steroid solutions (tobramycin and dexamethasone ophthalmic suspension 2-3 drops twice a day for 5 days) was also given. The patient's total hospital stay lasted 1 day.

**Figure 5:**
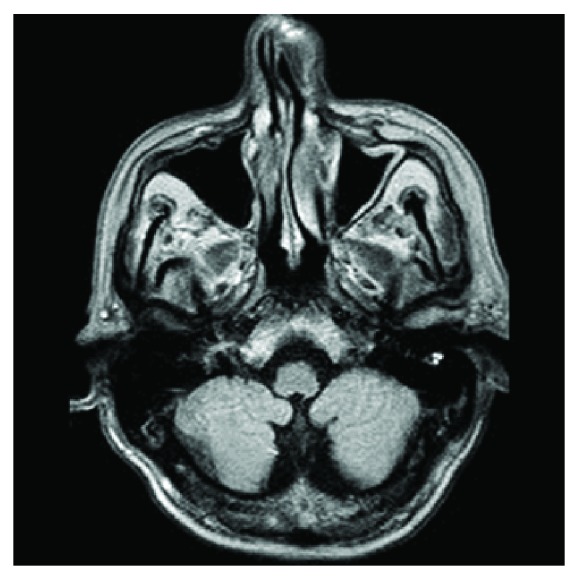
MRI scan 6 months after surgery.

**Figure 6:**
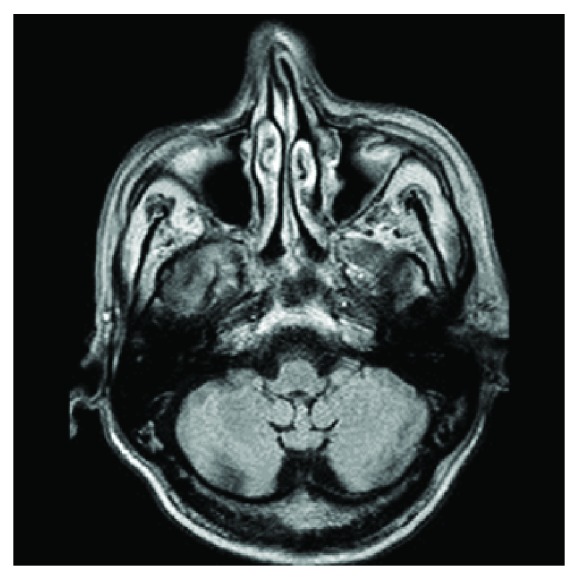
MRI scan 6 months after surgery.

A lacrimal duct system irrigation with natural saline solution into the inferior canaliculus was performed by an ophthalmologist once a week during the first month and twice a month during the second month. An endoscopic endonasal examination to test the patency of the duct system was performed 15, 21, and 30 days postoperatively. The silicone stent was removed 6 weeks postoperatively. An anatomical and functional obstruction of the DCR-window due to granuloma formation at the DCR-window area was noted 8 weeks after surgery on the combined nasal endoscopic and ophthalmologic examination. An endoscopic revision-DCR procedure with granuloma removal under local anaesthesia was performed. The DCR-window remained functionally and anatomically patent 6 months after surgery (Figures 5 and 6).

## 3. Discussion

Osteomas are slow growing benign tumors of bone, occurring most commonly in the craniofacial skeletal structures, mainly in the nasal and paranasal cavities. They usually develop at the frontal and ethmoid sinuses (90%), with the maxillary and sphenoid sinuses as well as the nasal cavity being rare sites of occurrence (10%) [3]. The cause of an osteoma is usually unidentified, but commonly accepted theories propose embryological, traumatic, or infective causes [4].

Some possible causes of a maxillary sinus osteoma include trauma, previous surgery, inflammation, or developmental abnormalities. Given that there was no history of previous trauma, paranasal surgery, or chronic sinusitis for this particular patient, the cause of osteoma formation is unclear.

In their early stages, osteomas of the paranasal sinuses are usually asymptomatic and are incidentally detected on imaging examinations for other conditions. Symptoms vary depending on tumor size and location. The most common symptoms include headache, facial pain, and nasal obstruction. Osteomas causing nasolacrimal duct obstruction with symptoms of epiphora or dacryocystitis are usually associated with the ethmoid sinus. A review of osteomas causing epiphora is summarized on Table 1.

In asymptomatic cases, excision is not always indicated and should be considered when the osteoma is responsible for occurring symptoms, if medical treatment of the symptoms is unsuccessful. In cases of osteoma extension beyond the sinus margins, surgical treatment is mandatory. Surgical approaches include external, endoscopic, and combined endoscopic and external procedures.

In cases where a sinus osteoma causes obstruction of the nasolacrimal duct and in the management of the diseases of the lacrimal system in general, close cooperation between the ENT surgeon and the ophthalmologist is of major importance. The combined nasal endoscopic and ophthalmologic examination, before and after the excision of the osteoma and the treatment of the obstruction, will ensure the functional and anatomic success.

## Figures and Tables

**Table 1:** Osteomas causing epiphora—review of the literature.

Reference			Age	Sex	Osteoma location	Symptoms
[5]	1984	Sternberg and Levine	15	N/A^1^	Ethmoid sinus	Epiphora
[3]	1999	Mansour et al.	66	F^2^	Ethmoid sinus	Orbital cellulitis, epiphora
[6]	2003	Osma et al.	35	M^3^	Ethmoid sinus	Epiphora
[7]	2003	Lin et al.	73	M	Middle turbinate	*Ι*psilateral facial pain, epiphora, nasal obstruction
[8]	2005	Kandogan et al.	39	F	Ethmoid sinus	Epiphora, facial pain
[9]	2005	Zouloumis et al.	34	F	Ethmoid sinus	Headache, epiphora, proptosis
[10]	2006	Lachanas et al.	16	M	Ethmoid sinus	Epiphora, mild pain in area of median canthus
[11]	2010	Daneshi et al.	41	F	Middle turbinate	Nasal obstruction, epiphora, nasal discharge
[12]	2013	Kim and Kwon	49	M	Nasolacrimal duct	Ipsilateral ocular pain, epiphora, medial canthal swelling

^1^N/A: not available.

^2^F: female.

^3^M: male.
